# Whole-Genome Sequence of *Leuconostoc mesenteroides* LT-38, a Non-Spore-Forming Gram-Positive Lactic Acid Bacterium

**DOI:** 10.1128/genomeA.00670-17

**Published:** 2017-08-03

**Authors:** Shiro Kato, Tadao Oikawa

**Affiliations:** a High Technology Research Core, Kansai University, Suita, Osaka, Japan; b Faculty of Chemistry, Materials, and Bioengineering, Kansai University, Suita, Osaka, Japan

## Abstract

The present study reports the complete genome sequence of *Leuconostoc mesenteroides* strain LT-38, which is a non-spore-forming Gram-positive lactic acid bacterium. The genome is composed of a 2,022,184-bp circular chromosome and contains 2,005 putative protein-coding genes.

## GENOME ANNOUNCEMENT

*Leuconostoc mesenteroides* is a non-spore-forming, Gram-positive, coccus-shaped lactic acid bacterium that is often found in a fermentative environment, including in fermented foods. Lactic acid bacteria produce bioactive molecules in food (1), and some strains of *L. mesenteroides* also produce functional compounds. For example, strain W3 isolated from wine (2) and strain 213M0 from Mongolian fermented mare milk (3) produce bacteriocin and a bacteriocin-like substance, respectively. Strain IFI-CA141, isolated from aged wine, produces biogenic amines, such as putrescine (4). Strain LK-151 (5), derived from Kimoto, a starter of the traditional brewing method of Japanese sake, produces a large amount of d-amino acids, which affects the taste of sake (6). Genome information on various *L. mesenteroides* strains will help us understand their potential productivities of various functional compounds and the strains will increase the quality and function of food.

The present report provides the complete genome sequence of *L. mesenteroides* LT-38, which was analyzed using a GS Junior 454 sequencer (Roche) for shotgun and paired-end sequencing and an ABI 3730 DNA analyzer (Thermo Fisher Scientific) for Sanger sequencing. Strain LT-38 is a type strain that is available from a public culture collection (National Institute of Technology and Evaluation, Japan) as strain NBRC 3426.

*L. mesenteroides* LT-38 was cultivated in de Man-Rogosa-Sharpe (MRS) broth, and the genomic DNA, with sufficient purity for the preparation of a DNA library, was extracted using a DNeasy blood and tissue kit (Qiagen). A whole-genome shotgun library was constructed using a GS Titanium rapid library preparation kit (Roche). An 8-kb-span paired-end library was also prepared using a GS Titanium rapid library preparation kit (Roche) and GS Titanium libraries of paired-end adaptors (Roche), according to Roche’s paired-end library preparation method. The GS De Novo Assembler version 2.9 assembled the whole-genome shotgun reads and paired-end reads into one scaffold, with approximately 67-fold genome coverage of the 2.0-Mb genome, and the chromosomal scaffold was composed of 38 large contigs. No scaffold for the plasmid was detected, which is different from *L. mesenteroides* ATCC 8293 (7) and *L. mesenteroides* J18 (8), and this is a characteristic feature of the LT-38 genome. The complete chromosomal length of strain LT-38, after filling the remaining gaps by Sanger sequencing, was 2,022,184 bp, with a G+C content of 37.56%. Putative coding sequences on the chromosomal DNA were predicted and annotated using the Microbial Genome Annotation Pipeline (9), and 2,005 genes encoding proteins, 68 genes for tRNAs, and 9 genes for rRNAs were detected. This genome information will facilitate an understanding of the whole metabolic capacity not only of strain LT-38 but also of other *L. mesenteroides* strains.

### Accession number(s).

The complete genome sequence has been deposited in DDBJ under the GenBank accession number AP017935.
